# Properties of Potato Starch Roasted with Apple Distillery Wastewater †

**DOI:** 10.3390/polym12081668

**Published:** 2020-07-27

**Authors:** Tomasz Zięba, Dominika Solińska, Małgorzata Kapelko-Żeberska, Artur Gryszkin, Jurislav Babić, Đurđica Ačkar, Francisca Hernández, Ante Lončarić, Domagoj Šubarić, Antun Jozinović

**Affiliations:** 1 Department of Food Storage and Technology, Faculty of Food Science, Wroclaw University of Environmental and Life Sciences, Chełmońskiego 37, 51-630 Wrocław, Poland; tomasz.zieba@upwr.edu.pl (T.Z.); dominika.solinska@upwr.edu.pl (D.S.); malgorzata.kapelko@upwr.edu.pl (M.K.-Ż.); artur.gryszkin@upwr.edu.pl (A.G.); 2 Faculty of Food Technology Osijek, Josip Juraj Strossmayer University of Osijek, Franje Kuhača 18, 31000 Osijek, Croatia; jbabic@ptfos.hr (J.B.); dackar@ptfos.hr (Đ.A.); ante.loncaric@ptfos.hr (A.L.); 3 Department of Plant Sciences and Microbiology, Miguel Hernández University, Ctra. de Beniel, km 3,2, 03312 Orihuela (Alicante), Spain; francisca.hernandez@umh.es; 4 Faculty of Agrobiotechnical Sciences Osijek, Josip Juraj Strossmayer University of Osijek, Vladimira Preloga 1, 31000 Osijek, Croatia; dsubaric@fazos.hr

**Keywords:** potato starch, apple distillery wastewater, esterification, roasting, properties

## Abstract

This study aimed to produce starch esters by roasting potato starch with apple distillery wastewater at various temperatures and aimed to determine the effects of esterification conditions on selected properties of the modified preparations. Apple distillery wastewater was concentrated, mixed with starch (30 g of dry matter per 100 g of starch), dried, and roasted at temperatures of 110, 130 or 150 °C for 3 h. The resulting preparations were rinsed 30 times with a 60% ethanol solution, dried, and disintegrated. After that, the following analyses were performed: content of substituted acids (after alkaline de-esterification) with high performance liquid chromatography (HPLC); thermal characteristics with differential scanning calorimetry (DSC); swelling power and solubility in water at 80 °C; color changes with a colorimeter; rheology of the pastes based on the plotted flow curves; and the pastes’ resistance to amyloglucosidase. Starch treatments with apple distillery wastewater at 130 and 150 °C caused significant changes to its properties when compared to the control samples of native starch and starch roasted without wastewater, including: a lower temperature and heat of pasting, lower swelling power and solubility in water, darker color, higher resistance to amyloglucosidase, and the formation of pastes with a lower viscosity.

## 1. Introduction

Starch, as the main component of cereal grains and tubers, is one of the most abundantly available, inexpensive, and biodegradable polymers, commonly used in the food, pharmaceutical, textile, biomass energy, and chemical industries [1,2,3]. Furthermore, it is the most important source of metabolic energy in the human diet [4]. From a nutritional aspect, starch can be classified into three categories based on digestibility: rapidly digestible starch (RDS), slowly digestible starch (SDS), and resistant starch (RS) [5,6,7]. RS is starch that is not digested in the small intestine (of healthy individuals), and might therefore be partially fermented by the gut microflora in the large intestine [5,7,8,9].

**Figure 1:**
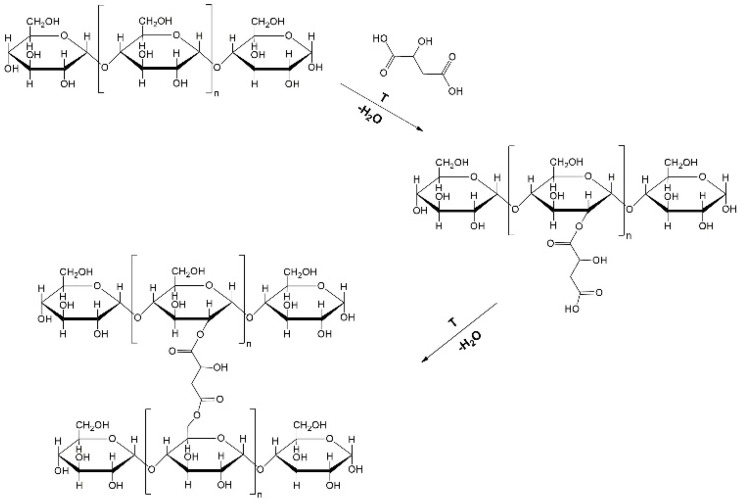
Mechanism of starch esterification by malic acid.

At present, different methods have been applied to prepare RS, including chemical, physical (hydrothermal), and enzymatic modification methods [10]. Among these techniques, the most effective way to increase the resistant starch content is through chemical modifications, such as carboxymethylation, esterification, and phosphorylation [4,5]. Recently, research on the reaction of starch and organic acids (citric, malic, lactic, acetic) in combination with heat-moisture treatment (HMT) as a means of manufacturing resistant starch in high yields has been increasingly favored [5,10,11,12]. During these reactions, the hydroxyl group on the glucose ring in the starch chain reacts with the organic acid in order to form a cross-linked structure with a high content of resistant starch (RS) [12]. One of the acids, which, in recent articles, has been the most popular for the esterification procedure, is malic acid [2,4,5,7,12]. The predicted reaction of starch and malic acid is shown in Figure 1, and it is combined with a high temperature to improve the reaction efficiency.

However, the use of chemical components in food production raises anxiety in a growing number of consumers. This is due to their increasing awareness of the risks and impacts such products have on the human body. The use, for starch esterification, of organic acids that occur naturally in plant materials may offer an alternative to its chemical modification. An example for this could be apple distillery wastewater, generally known as stillage or slops, which is a by-product of apple brandy production and which is generated in large quantities. It is wet (3–6% d. m.) and acidic waste (pH 3.5–3.9), in which various organic acids (malic, tartaric, citric, succinic, lactic) have been identified [13,14]. Along with other wastewaters from the food industry, this by-product represents a great ecological problem [15] because of the fact that food industry wastewaters are characterized by significant values of Biological and Chemical Oxygen Demand concentrations (BOD and COD, respectively). Therefore, the literature has reported a wide range of technologies and techniques for the treatment of food industry wastewaters [16]. So far, to our knowledge, no research has been published on the application of apple distillery wastewater in the starch modification process. In the investigation, potato starch was used; it has been classified as part of middle or high glycemic index (GI) foods, since it contains a high content of rapidly digestible starch (RDS) [17]. Because of the abovementioned, the aim of this study was to examine the possibility of using apple distillery wastewater in combination with roasting to produce potato starch esters with an increased content of RS, as well as to determine the effects of different esterification conditions on the selected properties of modified starch, which is important for its use in the food industry.

## 2. Materials and Methods

### 2.1. Materials

The initial study material was Superior Standard potato starch produced by PEPEES S.A. (Łomża, Poland) in 2018 and apple distillery wastewater obtained as a by-product after the distillation of fermented apple juice in a laboratory distillation device (ILMA, Požega, Croatia) at the Faculty of Food Technology Osijek.

### 2.2. Modification Procedure

Apple distillery wastewater was concentrated (to 50 °Bx), mixed with potato starch (30 g of dry matter per 100 g of starch), dried at 35 °C for 12 h, and then roasted at temperatures of 110, 130 or 150 °C for 3 h in a laboratory air dryer (Memmert, Büchenbach, Germany) in a thin layer with occasional stirring. The resulting preparations were rinsed 30 times with a 60% ethanol solution, dried, and disintegrated. Native starch was also roasted without apple distillery wastewater with the application of the same roasting procedure. The modified preparations were named: R 110 °C, R 130 °C, R 150 °C—roasted starch without wastewater, and RW 110 °C, RW 130 °C, RW 150 °C—roasted starch with wastewater.

### 2.3. Qualitative and Quantitative Analysis of Organic Acids by HPLC

The chromatographic analysis of organic acids was performed after refining the starch esters and after their alkaline de-esterification, which allowed for the assumption that all free acids were removed from the sample. Namely, a dose of starch roasted with wastewater (20 g per dry matter basis) was transferred to a conical flask with 300 mL of distilled water. The flask was placed on a magnetic stirrer, and the suspension was brought to the boiling point and then stirred for 10 min. After cooling, 1 L of rectified ethanol was added to the flask to enable starch precipitation. The sample was left for 24 h, and the excess of clear solution was decanted from above the precipitate. The refining process (dissolving in water and precipitation with ethanol) was carried out three times. Finally, the precipitated starch was dried in an air dryer at 35 °C, milled, and sieved through a 400-mm sieve.

Afterwards, alkaline de-esterification was conducted. For this purpose, 2 g of the refined starch sample (per dry matter basis) and 100 mL of 0.5 M NaOH were mixed in a magnetic stirrer at a temperature of 35 °C for 12 h. Then, 400 mL of rectified ethanol was added to the homogenous solution to enable starch precipitation. The obtained filtrate was concentrated by evaporation on an evaporator (BÜCHI Labortechnik AG, Flawil, Switzerland) to a volume of 10 mL. Next, a 1-mL aliquot of the concentrated solution was filtered through a Millipore membrane filter with a mesh size of 0.45 μm and analyzed with the technique of high-performance liquid chromatography (HPLC) (Hewlett-Packard series 1100; Hewlett-Packard, Wilmington, DE, USA) according to the method of Hernández et al. [18]. The analysis was performed using a 0.1% buffer for elution with orthophosphoric acid, at a flow rate of 0.5 mL min^−1^, using a Supelcogel TM C–610H column (30 cm × 7.8 mm) with a pre-column (Supelguard 5 cm × 4.6 mm; Supelco, Inc., Bellefonte, PA, USA). The absorbance was measured at a wavelength of 210 nm with a diode array detector (DAD). Calibration curves (*R*^2^ = 0.9990) were plotted in triplicates, using organic acid standards by Sigma-Aldrich (Poole, UK). The degree of substitution was expressed as the percentage content of acid residues in the preparations, according to Zdybel et al. [19].

In the case of apple distillery wastewater, it was only centrifuged for the analysis, and after that, 1 mL of aliquot was passed through a 0.45 μm Millipore filter and was then used for the same HPLC procedure. The results were expressed as the concentration in g per 100 mL of sample.

### 2.4. Determination of Swelling Power and Solubility in Water

A water suspension was prepared from a starch preparation that contained 1 g of starch per 100 g of solution. The suspension was shaken at a temperature of 80 °C for 30 min. Afterwards, the sample was cooled to 20 °C and centrifuged for 30 min using a Biofuge 28RS centrifuge (Heraeus Sepatech, Hanau, Germany) with an acceleration of 22,500× *g*. The separated supernatant was determined for the dry matter content with the gravimetric method at 105 °C, and the precipitate left in the centrifuge tubes was weighed [20].

### 2.5. Determination of Thermal Characteristics

Thermal properties were determined with a differential scanning calorimeter DSC 822E (Mettler Toledo, Giessen, Germany), following the methodology provided by Gryszkin et al. [21]. Before the measurement, the calorimeter was calibrated using a sample of indium and a sample of zinc. The starch preparation (10 mg on a dry matter basis) was weighed into ME-51119871 medium-pressure crucibles, and redistilled water was added at a ratio of 3:1 (water:starch). Afterwards, the crucible was sealed and conditioned at a temperature of 25 °C for 30 min. The analysis was carried out in a temperature range of 25–100 °C at a heating rate of 4 °C/min.

### 2.6. Determination of Flow Curves of Pastes

For the determination, the prepared suspensions contained 5 g of starch per 100 g of solution and were then heated at a temperature of 96 °C for 30 min with continuous mixing. The analysis was performed using a Haake Rheostress 6000 universal rheometer (Thermo Fisher Scientific Inc., Karlsruhe, Germany), according to the method of Gryszkin et al. [22]. The flow curves of pastes were determined in a set of coaxial (Z32AL) cylinders at 50 °C, with a shear rate range of 1–300 s^−1^. The flow curves were described by the following equations [23]:

(1) $${\mathrm{Ostwald}}^{\prime }s:\;\;\;\;\;\;\tau =K\cdot {\dot{\gamma }}^{n}$$

(2) $${\mathrm{Casson}}^{\prime }s:\;\tau^{0.5}=\tau_{0C}{}^{0.5}+{\left(\eta_{C}\cdot \dot{\gamma }\right)}^{0.5}$$

where: τ—shear stress (Pa), $\dot{\gamma }$—shear rate (s^−1^), K—consistency coefficient (Pa s^n^), τ_0_ (τ_0C_)—yield stress (Pa), η_C_—Casson’s plastic viscosity (Pa s), n—flow behavior index.

### 2.7. Determination of Resistance to Amyloglucosidase Activity

The prepared suspensions contained 0.36 g of starch per 100 g of solution. They were heated to the boiling point with continuous mixing and then cooled to a temperature of 37 °C, at which point hydrolysis with amyloglucosidase (Amigase by Genecor) (Danisco, Copenhagen, Denmark) was conducted. Acetate buffer (pH = 4.5) was added to the prepared suspension of the starch preparation at a ratio of 1:1. The flask was placed in a water bath with a shaker at a temperature of 37 °C (Memmert, Büchenbach, Germany), and 4 mL of an enzyme solution was added (at an enzyme-to-buffer ratio of 1:4). The concentration of the enzyme was selected so as to enable the complete saccharification of gelatinized starch after 120 min of the process. Every hour, 1 mL of the hydrolysate was collected and mixed with 95% ethanol (20 mL) to stop enzymatic digestion. These solutions were centrifuged (MPW Instruments, Poland) at a speed of 1825× *g* for 5 min. The supernatant was collected from the centrifuged sample and mixed with the reagent GLUCOSE (containing glucose oxidase and peroxidase) from the Biosystem company (Barcelona, Spain), and was then incubated at 20 °C for 15 min. Afterwards, the absorbance was measured using a CECIL CE 2010 colorimeter (Cecil Instruments, Cambridge, UK) at a wavelength of λ = 500 nm. The measurements were conducted against a blank sample constituted by a reagent with an acetate buffer. The quantity of glucose was read out from a standard curve that was plotted as above using glucose (p.a.) solutions. The degree of saccharification was computed with respect to the theoretical quantity of glucose produced from the complete saccharification of a weighted portion of starch. The result of the hydrolysis was considered final when three consecutive read-outs of the absorbance did not differ from one another [23].

### 2.8. Determination of the Color Difference

The color difference (darkening) was calculated from the results obtained in the CIE-Lab system by measuring L* (whiteness/darkness), a* (redness/greenness), and b* (yellowness/ blueness). The measurement was performed with a Chroma Meter CR-200 (Konica Minolta, Tokyo, Japan) by using granular materials attachment. The total color change (ΔE) was calculated with the following equation [24]:

(3) $$\Delta E={\left(\Delta L^{2}+\Delta a^{2}+\Delta b^{2}\right)}^{0.5}$$

where the difference of the parameters was calculated with reference to the native potato starch.

### 2.9. Statistical Analysis

The results obtained in the study were analyzed statistically using the software STATISTICA 13.3 (StatSoft, Inc., Tulsa, OK, USA). Based on the statistical calculations (from at least three parallel repetitions), the Duncan’s least significant differences (LSD) test was determined for selected relationships at *p* < 0.05 and standard deviations.

## 3. Results and Discussion

Cider is an alcoholic beverage made from apple juice, which, in addition to ethanol, contains: unfermented sugars, glycerol, organic acids [25], and small amounts of phenolic compounds [26], fatty acids [27], and easily volatile compounds that define the aroma of the drink [28]. The wastewater obtained during the production of brandy is free of ethanol and volatile compounds. In the experiment we carried out, substances in the distillery wastewater–organic acids may, as a practical matter, be relevant as starch modifiers. In cider, malic acid is present in the greatest quantity, and other organic acids are present in much smaller quantities, and their content depends on the type of raw material and the method of fermentation [29].

The concentrated apple distillery wastewater (used in the experiment) contained 15.96 g/100 mL of malic acid, 3.34 g/100 mL of lactic acid, and trace amounts of succinic, malonic, and oxalic acids (results not presented).

The content of organic acids in fruit varies widely and depends, among other things, on the type, variety, degree of ripeness, vegetation conditions, time, and storage conditions [30]. Most fruit is mainly rich in malic acid, which is the predominant organic acid in apples [31] or pears [32] and which, on average, accounts for up to about 85% of all organic acids [31].

**Figure 2:**
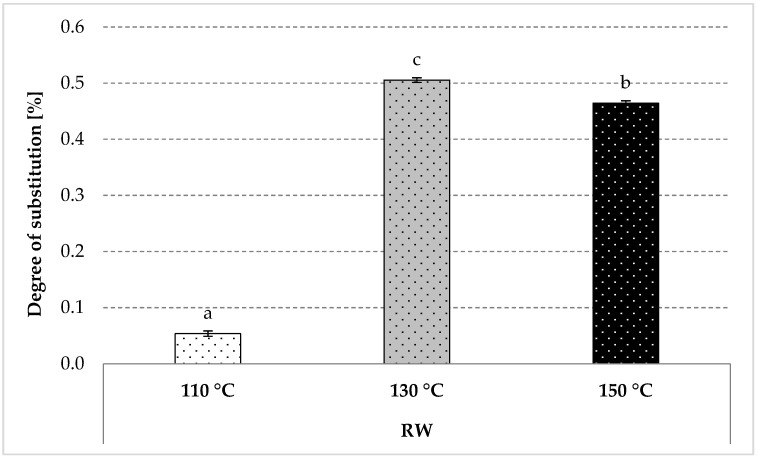
Degree of substitution determined in the modified starch preparations roasted with apple distillery wastewater. The results are expressed as the mean ± standard deviation (SD) (*n* = 3). Different letters above the bars indicate that the values are significantly different at *p* < 0.05. RW—roasted starch with wastewater.

Starch roasting with apple distillery wastewater allowed for the production of a starch ester mainly substituted by malic acid residues and trace amounts of other acids (Figure 2). The heating at the lowest tested temperature resulted in a negligible starch substitution (0.05 g/100 g), whereas the roasting at a higher temperature led to a ten-fold higher degree of substitution, with a more significant effect obtained for starch roasted at 130 °C (Figure 2). The impact of the roasting temperature on the course of starch esterification with organic acids has been extensively discussed in the literature [33,34]. So far, pure acid solutions have been used for esterification, while the novelty of the present study lies in starch modification with a natural waste material from apple brandy production.

**Figure 3:**
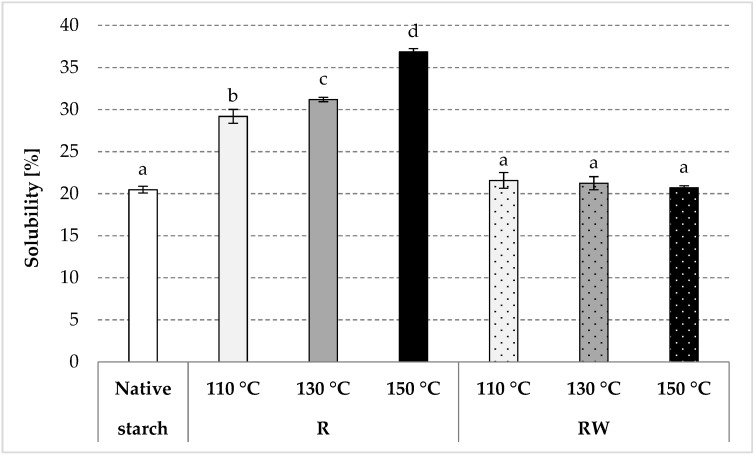
Solubility in water determined in the native and modified starch preparations. The results are expressed as the mean ± SD (*n* = 3). Different letters above the bars indicate that the values are significantly different at *p* < 0.05. R—roasted starch without wastewater; RW—roasted starch with wastewater.

**Figure 4:**
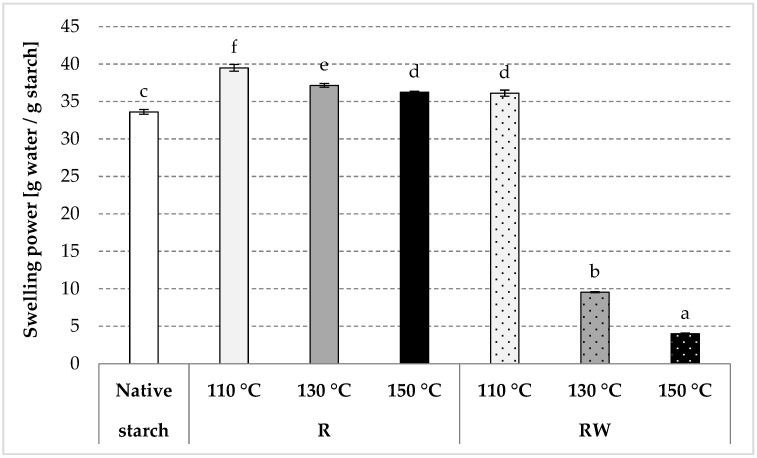
Swelling power determined in the native modified starch preparations. The results are expressed as the mean ± SD (*n* = 3). Different letters above the bars indicate that the values are significantly different at *p* < 0.05. R—roasted starch without wastewater; RW—roasted starch with wastewater.

As a result of roasting, native starch undergoes thermal depolymerization and transglycosidation [35]. Consequently, the water solubility of starch increases, and its swelling power decreases as the process temperature increases [34,36]. An analogous tendency was observed in the present study due to native starch (R) roasting at various temperatures (Figure 3 and Figure 4). Starch esterification with apple distillery wastewater resulted in values of starch solubility in water of ca. 21% for all preparations, similar to the values obtained for native potato starch (Figure 3). On the other hand, we determined a few-fold decrease (to a few g/g) in the swelling power of esters produced by roasting with wastewater at 130 or 150 °C (Figure 4). The roasting at the lower temperature caused a smaller loss of the swelling power, presumably due to a low degree of substitution of the modified preparation. The esterification of starch carried out in the experiment, despite its relatively low substitution (up to about 0.5%), caused unexpectedly large changes in the discussed starch characteristics. The effect of a negligible (about 0.1%) natural esterification of potato starch with residual phosphoric acid on the properties of the prepared glues is commonly known [37]. It should also be remembered that even the smallest differences in the chemical structure (e.g., amylose content [38], degree of polymerization [39]), physical structure (e.g., porosity of the surface of the starch granules [40], its size [41], degree of crystallinity [42]), or the presence of other substances (e.g., protein, fat [43], this being an integral part of the starch granules) factor significantly into starch properties [37,44]. However, the addition of even small amounts of salts, sugars, or hydrocolloids causes significant changes in the rheological properties of prepared starch pastes [45,46]. Shi et al. [5] concluded that starch esterification with malic acid causes a significant decrease in both its water solubility and swelling power. Changes in these properties are determined by the reagent type and conditions of the modification process [47,48,49,50,51,52,53,54,55,56,57,58,59]. In addition, starches with a low degree of substitution are usually characterized by a higher solubility in water and a higher swelling power when compared to starch with a high substitution degree [60,61,62,63,64,65]. A decrease in the values of the discussed properties is also attributable to starch crosslinking [66], which leads to the production of a water-insoluble preparation from starch with a high degree of substitution [37,67,68,69].

The thermal characteristics of gelatinization depend on the roasting temperature of starch [70]. The characteristic temperature range for this process is from 60 to 70 °C, and the heat of the phase transition reaches approximately 17 J/g on average [71,72]. In both cases (with or without wastewater), the roasting of native starch caused a decrease of all gelatinization parameters, with the exception of starch roasted with apple distillery wastewater at 130 °C. In the case of starch roasted without wastewater (R), various temperatures caused negligible changes in the thermal characteristics of gelatinization (Table 1), which were attributable to the reduction of the initial gelatinization temperature and to a slight increase in the heat of the phase transition along with a roasting temperature increase. A similar tendency was reported by Kapelko-Żeberska et al. [34] in a study on starch roasted with citric acid. In the present study, the produced starch esters were capable of paste formation. Starch roasting with apple distillery wastewater (RW) caused changes in the initial and final temperatures of gelatinization, which depended on the degree of substitution (Table 1). It was noticed that the initial gelatinization temperature decreased along with an increasing degree of esters substitution. When compared to the roasted native starch without wastewater, the ester that was obtained by roasting starch with apple distillery wastewater at the lowest tested temperature (RW 110 °C) was characterized by a higher initial gelatinization temperature, whereas the ones obtained by roasting at the higher temperatures were characterized by a lower initial gelatinization temperature. In turn, the ester of the starch roasted with the apple distillery wastewater at 130 °C was characterized by the lowest initial gelatinization temperature due to the highest degree of esterification. An analogous tendency was observed for the final temperature of gelatinization.

**Figure 5:**
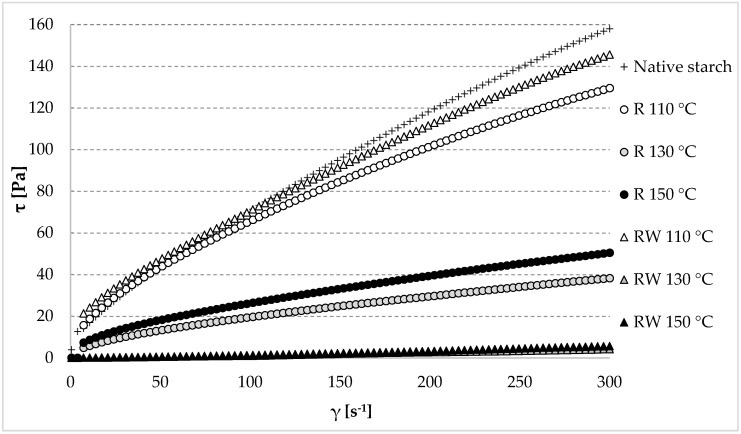
Flow curves of the native and modified starch preparations. The results are expressed as the mean (*n* = 3). R—roasted starch without wastewater; RW—roasted starch with wastewater.

Considering the functionality of starch preparations, the rheological properties of pastes that are made from them play a key role in the food production process. The obtained results are presented in the form of flow curves (Figure 5). It is evident from the obtained results that the lowest changes were caused by roasting at a temperature of 110 °C, as in the cases both with or without wastewater.

Table 2 provides the values of the consistency coefficient (K), this being a measure of the paste viscosity at the initial stage of shearing [72,73], as well as the values of the yield stress (τ_oc_) and plastic viscosity (η_c_), described with the Casson’s model. The yield stress is a measure of the maximum paste stress at the null shear rate, whereas Casson’s plastic viscosity indicates the paste viscosity at the final stage of shearing [21]. Among the starch pastes prepared from roasted native starch without wastewater (R), the highest viscosity in the entire course of the flow curve was demonstrated for the one made of native starch roasted at the lowest temperature (R 110 °C). The pastes made of native starch roasted at the higher tested temperatures (R 130 °C or R 150 °C) were characterized by a significantly lower viscosity (Figure 5). Analogous tendencies were observed for the rheological coefficients (Table 2). Presumably, this was due to the progressing thermolysis of starch and, consequently, to the lower viscosity of the pastes. The impact of the molecular weight of starch on the viscosity of pastes was reported by Praznik et al. [74]. The paste made of native starch roasted with apple distillery wastewater at the lowest analyzed temperature (RW 110 °C) was characterized by the highest viscosity in the whole course of the flow curve (Figure 5). An increase in viscosity was probably due to the esterification reaction at the relatively small thermo-acidic hydrolysis of starch. The roasting at the higher tested temperatures (RW 130 °C or RW 150 °C) caused a drastic decrease in the paste viscosities in the entire course of the flow curve, which could have been caused by the acidic hydrolysis and thermolysis of starch proceeding in these conditions despite the high esterification degree. An analogous tendency was observed for the rheological coefficients (Table 2). Due to the low viscosity of the pastes prepared from the esters obtained at 130 or 150 °C, it turned out to be impossible to determine the values of the rheological coefficients described according to Casson’s model (Table 2), whereas the value of the flow coefficient n determined from the Ostwald de Waele model was higher than 1, which is not typical for starch pastes. The other starch pastes made of the native and roasted native starch (R) and of the ester produced at the lowest temperature with apple distillery wastewater exhibited properties of shear-thinned non-Newtonian fluids (Table 2). Many literature sources claim that this rheological character of starch pastes is typical [75,76,77,78,79,80].

**Figure 6:**
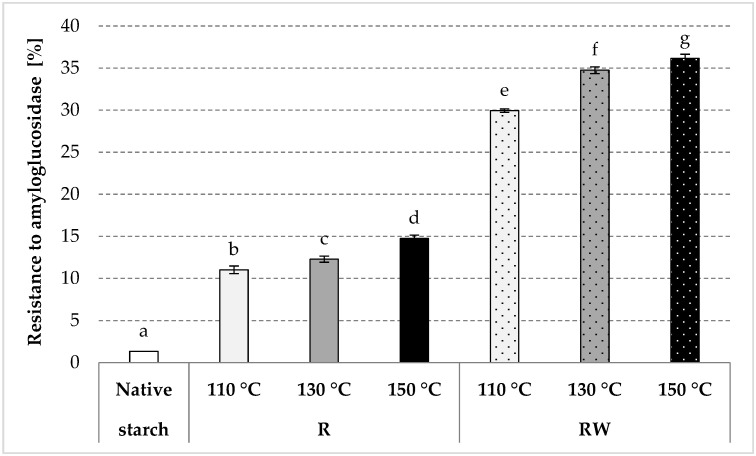
Resistance to amyloglucosidase determined in the native and modified starch preparations. The results are expressed as the mean ± SD (*n* = 3). Different letters above the bars indicate that the values are significantly different at *p* < 0.05. R—roasted starch without wastewater; RW—roasted starch with wastewater.

The resistance of roasted native starch to the activity of amyloglucosidase increased significantly in comparison to the native potato starch, with a more significant impact at higher roasting temperatures (Figure 6), which was due to the repolymerization occurring during starch roasting [81]. The esters produced by native starch roasting with apple distillery wastewater revealed a significantly higher resistance when compared to the samples roasted without wastewater. The resistance was observed as increasing along with the increasing temperature of roasting, whereas the starch roasted at the highest temperature was characterized by an enzymatic resistance of approximately 36%. A pea starch ester with malic acid, which has a significantly higher degree of substitution (DS = 0.146, which corresponds to ca. 10 g of acid residues/100 g preparation) than the esters produced in this study, contains about 41% of RS [5]. The resistance of RS4 type starch depends mainly on the type of chemical modification and the substitution degree [81]. Starch resistance increases along with the increase in the substitution degree [82]. However, like in the case of the properties described earlier, the resistance of the produced esters was mostly determined by the roasting temperature. A similar conclusion was drawn by Kapelko-Żeberska [34] in their study on the production of resistant starch by roasting potato starch with citric acid. The resistance of acetylated retrograded starch preparations to amylolysis is determined not only by the total degree of substitution but also by the substitution at the carbon atoms 2 and 3 neighboring the 1,4-glycosidic bond that is being hydrolyzed [83]. From the perspective of food technology, the resistant starch preparations analyzed in this study were not produced via chemical modification because they were obtained from natural raw materials without a chemical reagent. For this reason, they do not have to be treated as food additives when considering their potential industrial application.

According to the results obtained for the color, it is evident that all the measured parameters changed significantly with the increase of the roasting temperature (Table 3). Higher temperatures caused a darkening of the samples, which could be concluded from the decrease in the L* values with an increasing temperature. Compared to the native starch and starch roasted without wastewater, the changes in the color of the starch roasted with apple distillery wastewater were significantly higher. This was probably due to the caramelization reaction that proceeded with high intensity in the acidic medium [84]. It is important to emphasize that, in the samples roasted with apple distillery wastewater, the b* values (yellowness) were higher and the a* values were in positive, which indicates the presence of a red hue in these starches. The roasting of starch without wastewater caused a negligible total color change (ΔE) in comparison with native potato starch, while these changes were significantly higher in the starch roasted with apple distillery wastewater. The higher roasting temperatures led to significant changes in the color of the produced preparations, with the most significant effect occurring at a temperature of 130 °C.

## 4. Conclusions

The study results lead to the conclusion that it is feasible to produce starch esters via the roasting of native potato starch with a natural waste product—in this case, apple distillery wastewater—and that the properties of the modified preparations predispose them for use in the food industry, as well as in pharmacy or medicine. Namely, it is known that esterified starches have various applications, such as being used as thickeners, additives, stabilizers, emulsifiers, flavoring ingredients, or as fillers, coatings, binders, etc. The preparation obtained by roasting at 110 °C, characterized by an approx. 30% resistance to amyloglucosidase activity and the formation of pastes with a relatively high viscosity, could be used as a novel type of resistant starch with texture-forming properties in the production of new food products. Finally, it is important to emphasize that the obtained results represent a significant scientific contribution, since no research to date has been published on apple distillery wastewater application in starch modification processes. The production of resistant starch (RS) using natural raw materials combined with physical modifications could have a positive effect on the health-promoting value of food products and, in doing so, would satisfy consumer expectations. This research is part of one project: in the future we plan to investigate other similar by-products from the food industry for potential applications in the modification procedures of different starches. Furthermore, many other conditions and analyses will be included in order to obtain a better insight into the properties and potential applications of obtained starches.

## Figures and Tables

**Table 1:** Parameters of the phase transition of the native and modified starch preparations determined from thermal characteristics (DSC).

Sample		Initial Temperature [°C]	End Temperature [°C]	Heat of Transition [J/g]
**Native starch**		56.93 ± 0.03 ^f^	69.27 ± 0.02 ^c^	13.26 ± 0.41 ^a, b^
**R**	**110 °C**	55.61 ± 0.20 ^e^	68.13 ± 0.39 ^b^	12.14 ± 0.21 ^a^
	**130 °C**	54.58 ± 0.09 ^d^	67.39 ± 0.16 ^a, b^	12.81 ± 0.52 ^a, b^
	**150 °C**	54.29 ± 0.27 ^c^	67.88 ± 0.52 ^b^	13.56 ± 1.34 ^b^
**RW**	**110 °C**	59.00 ± 0.10 ^g^	70.66 ± 0.36 ^d^	12.10 ± 0.21 ^a^
	**130 °C**	51.97 ± 0.06 ^a^	66.84 ± 0.29 ^a^	12.44 ± 0.55 ^a, b^
	**150 °C**	53.56 ± 0.15 ^b^	67.52 ± 0.66 ^a, b^	12.77 ± 0.29 ^a, b^

The results are expressed as the mean ± standard deviation (SD) (*n* = 3). Values with different letters (^a–f^) in the same column are significantly different at *p* < 0.05. R—roasted starch without wastewater; RW—roasted starch with wastewater.

**Table 2:** Parameters of Ostwald’s and Casson’s model describing the flow curves of the native and modified starch preparations.

**Sample**		**Ostwald’s Model**		
		**K [Pa s^n^]**	**n**	**R^2^**
**Native starch**		4.1889 ± 0.0270 ^e^	0.6397 ± 0.0016 ^d^	0.9835 ± 0.0012 ^a^
**R**	**110 °C**	3.9073 ± 0.3026 ^d^	0.6122 ± 0.0120 ^c^	0.9998 ± 0.0001 ^d^
	**130 °C**	1.2867 ± 0.0350 ^b^	0.5948 ± 0.0017 ^b^	0.9996 ± 0.0001 ^c, d^
	**150 °C**	1.8697 ± 0.0586 ^c^	0.5731 ± 0.0014 ^a^	0.9993 ± 0.0001 ^c, d^
**RW**	**110 °C**	3.8730 ± 0.0324 ^d^	0.6351 ± 0.0007 ^d^	0.9987 ± 0.0001 ^c^
	**130 °C**	0.0051 ± 0.0003 ^a^	1.1770 ± 0.0030 ^e^	0.9974 ± 0.0005 ^b^
	**150 °C**	0.0031 ± 0.0000 ^a^	1.3170 ± 0.0000 ^f^	0.9997 ± 0.0000 ^d^
**Sample**		**Casson’s Model**		
		**τ_0C_ [Pa]**	**η_C_ [Pa s]**	**R^2^**
**Native starch**		11.0167 ± 0.1815 ^c^	0.2190 ± 0.0041 ^c^	0.9872 ± 0.0049 ^a^
**R**	**110 °C**	10.4920 ± 0.8342 ^c^	0.2254 ± 0.0072 ^c^	0.9986 ± 0.0005 ^b^
	**130 °C**	3.5380 ± 0.1047 ^a^	0.0640 ± 0.0011 ^a^	0.9992 ± 0.0001 ^b^
	**150 °C**	5.1363 ± 0.1609 ^b^	0.0777 ± 0.0019 ^b^	0.9993 ± 0.0001 ^b^
**RW**	**110 °C**	10.7533 ± 0.1102 ^c^	0.2634 ± 0.0029 ^d^	0.9998 ± 0.0000 ^b^
	**130 °C**	n. d.	n. d.	n. d.
	**150 °C**	n. d.	n. d.	n. d.

n. d.—not detected; The results are expressed as the mean ± SD (*n* = 3). Values with different letters (^a–f^) in the same column are significantly different at *p* < 0.05. R—roasted starch without wastewater; RW—roasted starch with wastewater.

**Table 3:** Color differences determined in the modified starch preparations.

Sample		L*	a*	b*	ΔE
**Native starch**		97.13 ± 0.02 ^g^	−0.24 ± 0.04 ^c^	1.58 ± 0.07 ^a^	-
**R**	**110 °C**	96.13 ± 0.02 ^f^	−0.27 ± 0.03 ^b, c^	1.85 ± 0.03 ^b^	1.04 ± 0.01 ^a^
	**130 °C**	96.09 ± 0.02 ^e^	−0.32 ± 0.04 ^b^	2.00 ± 0.05 ^c^	1.13 ± 0.03 ^b^
	**150 °C**	95.68 ± 0.02 ^d^	−0.43 ± 0.03 ^a^	3.60 ± 0.04 ^d^	2.49 ± 0.02 ^c^
**RW**	**110 °C**	88.58 ± 0.03 ^c^	1.75 ± 0.05 ^d^	9.31 ± 0.04 ^e^	11.70 ± 0.00 ^d^
	**130 °C**	72.33 ± 0.04 ^a^	5.02 ± 0.05 ^f^	15.59 ± 0.04 ^g^	28.97 ± 0.06 ^f^
	**150 °C**	74.95 ± 0.01 ^b^	4.55 ± 0.02 ^e^	15.16 ± 0.03 ^f^	26.44 ± 0.01 ^e^

The results are expressed as the mean ± SD (*n* = 3). Values with different letters (^a–g^) in the same column are significantly different at *p* < 0.05. R—roasted starch without wastewater; RW—roasted starch with wastewater.
